# Serosurvey of Chikungunya Virus in Old World Fruit Bats, Senegal, 2020–2022

**DOI:** 10.3201/eid3007.240055

**Published:** 2024-07

**Authors:** William M. de Souza, Alioune Gaye, El Hadji Ndiaye, Angelica L. Morgan, El Hadji Daouda Sylla, Faty Amadou SY, Mawlouth Diallo, Scott C. Weaver

**Affiliations:** University of Kentucky, Lexington, Kentucky, USA (W.M. de Souza);; University of Texas Medical Branch, Galveston, Texas, USA (W.M. de Souza, A.L. Morgan, S.C. Weaver);; Institut Pasteur de Dakar, Dakar, Senegal (A. Gaye, E.H. Ndiaye, F. Amadou SY, M. Diallo);; Institut de Recherche pour le Développement, Dakar (E.H. Daouda Sylla)

**Keywords:** Chikungunya virus, Old World fruit bats, Senegal, viruses, zoonoses, vector-borne infections

## Abstract

We conducted a cross-sectional serosurvey for chikungunya virus (CHIKV) exposure in fruit bats in Senegal during 2020–2023. We found that 13.3% (89/671) of bats had CHIKV IgG; highest prevalence was in *Eidolon helvum* (18.3%, 15/82) and *Epomophorus gambianus* (13.7%, 63/461) bats. Our results suggest these bats are naturally exposed to CHIKV.

Chikungunya virus (CHIKV) is a mosquitoborne alphavirus that has caused >10 million cases in >125 countries and territories in the past 2 decades. Chikungunya disease is characterized by acute and chronic signs and symptoms in humans and can sometimes lead to neurologic complications and fatal outcomes (*1*). CHIKV is transmitted among humans mainly by *Aedes aegypti* and *Ae. albopictus* mosquitoes in a human-amplified urban cycle (*2*). The virus is also transmitted in ancestral African enzootic cycles involving several species of arboreal mosquito vectors that transmit among diverse, nonhuman primates and possibly other amplifying hosts (*2*,*3*). The role of Old World fruit bats (Pteropodidae) in CHIKV transmission in West Africa remains understudied. We investigated CHIKV exposure of these bats in the Kédougou region in Senegal, a CHIKV-enzootic region with a history of spillover epidemics but not human-amplified, *Ae*. *aegypti*–borne outbreaks (A. Padane et al., unpub. data).

During October 23, 2020–March 4, 2022, we collected blood samples from fruit bats in 5 locations in the Kédougou region of southeastern Senegal (Figure, panel A). All bats were identified by external morphology. We tested all serum samples (dilution 1:100) in duplicate by an in-house ELISA for detection of IgG against CHIKV by using a recombinant envelope 2 protein and an anti-bat secondary antibody. We defined the cutoff value for positive results as the mean of negative controls (uninfected mice) plus 3 SDs (Appendix). All animals collected were adults and apparently healthy at the time of sampling. All procedures followed the approval of the National Ethical Committee for Research of Senegal and the University of Texas Medical Branch Institutional Animal Care and Use Committee.

We analyzed blood samples from 671 bats belonging to 13 species across 6 families. *Epomophorus gambianus* bats represented 68.7% (461/671) of captured specimens, followed by *Micropteropus pusillus* (13.1%) and *Eidolon helvum* (12.1%) bats. We detected IgG against CHIKV envelope 2 protein in 13.3% (89/671) of bats tested (Figure, panel B; Appendix Table). Testing revealed the bat species most frequently seropositive in 4 of 5 sites analyzed to be *E. helvum* (18.3%, 15/82), *E. gambianus* (13.7%, 63/461), and *M. pusillus* (8%, 7/88) (Figure, panel B; Appendix Figure). The locations with the highest seroprevalence were Ndebou (20.9%, 18/86) and Samecouta (18.4%, 58/316) (Figure, panel B). CHIKV seropositivity was consistent in bats collected in 2020 (13.7%, 17/124) and 2021 (13.2%, 72/325). Also, CHIKV seropositivity rates were similar between male (14.2%, 69/485) and female (13.2%, 19/144) bats.

**Figure:**
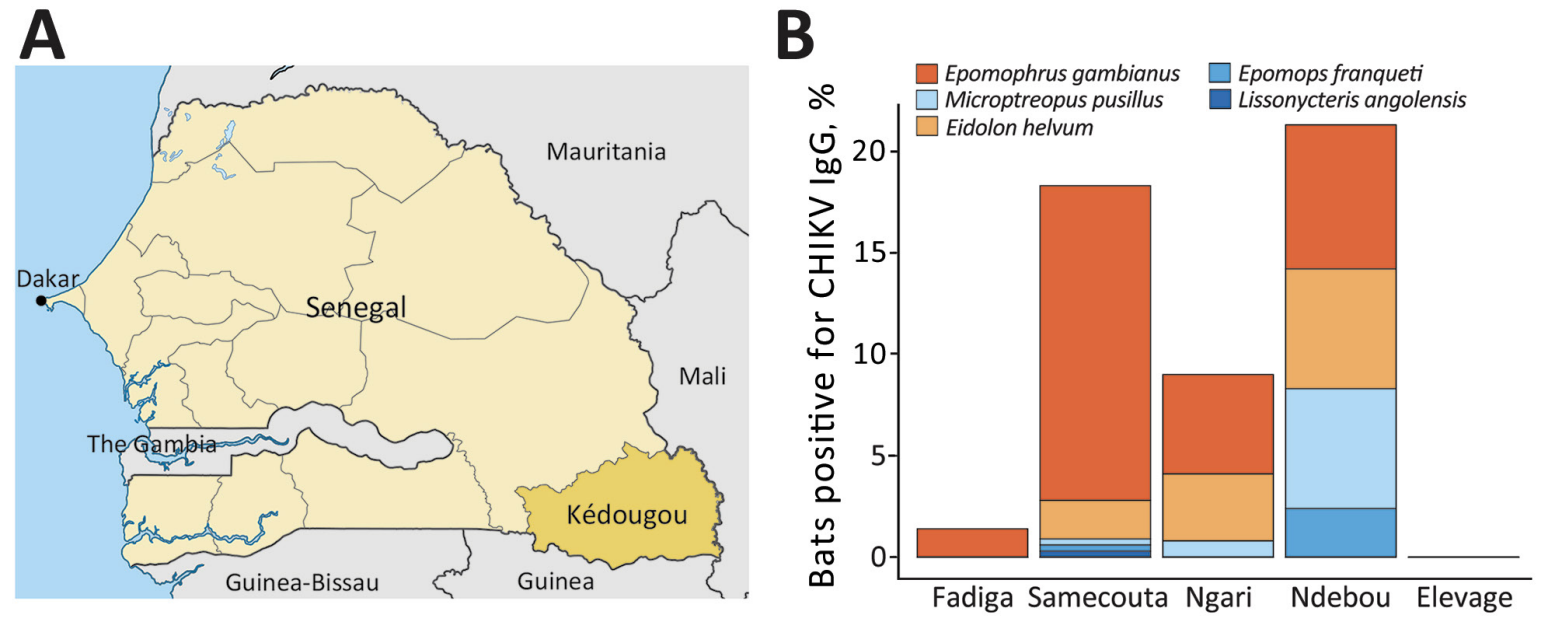
Serosurvey of CHIKV in the Kédougou region, Senegal. A) Location of Kédougou region (dark yellow) within Senegal (light yellow). B) Colored bars show the proportion of bats testing positive for CHIKV IgG at each capture site. Each color corresponds to a specific bat species, as indicated in the key above the graph. CHIKV, chikungunya virus.

We identified IgG specific for CHIKV in 5 species of fruit bats in several rural areas within the Kédougou region of southeastern Senegal before a 2023 outbreak (A. Padane et al., unpub. data). Bats are recognized to traverse wild, rural, and urban zones, and possess favorable biologic features for hosting and amplifying several emerging viruses, including viral spread across large geographic areas linked to migration (*4*). CHIKV has been previously isolated from *Scotophilus* spp. bats in Senegal (*5*). Experimental infection of *Eptesicus fuscus* bats with CHIKV demonstrated persistent viremia, followed by neutralizing antibody production without apparent clinical signs (*6*), compatible features for enzootic amplifying and reservoir host status. Of note, other domestic and wild animals (e.g., birds and livestock) appear unlikely to amplify CHIKV effectively (*6*). One study revealed that 36% (15/42) of fruit bats captured near human settlements tested positive for CHIKV after an initial outbreak in Grenada Island (*7*), suggesting that CHIKV can infect bats during human-amplified outbreaks. Another study found that 0.7% (2/303) of *Rousettus aegyptiacus* bats in Uganda have neutralizing antibodies against CHIKV (*8*).

Collectively, our findings suggest that *E. gambianus*, *E. helvum,* and *M. pusillus* bats are exposed to CHIKV infection in the enzootic cycle in West Africa. Limitations of our study include the absence of more specific neutralizing antibody tests in bat samples because of limited volumes of blood collected and the need for testing for antibodies against several other viruses. Thus, we recognize that some CHIKV-positive samples could have resulted from cross-reactions with other alphaviruses circulating in the region, particularly o’nyong-nyong virus (Appendix) (*9*). Nonetheless, *E. gambianus* bats, unlike highly migratory *E. helvum* bats, are rarely observed to migrate or disperse long distances. This fact suggests that the high seropositivity we noted is unlikely due to cross-reaction with o’nyong-nyong virus, a virus rarely detected in West Africa. Limited blood sample volumes also prevented molecular testing (e.g., reverse transcription PCR) to identify active CHIKV infections. Future investigations should prioritize direct virus detection and isolation from bats. In addition, although our serologic data indicate past exposure, we could not ascertain the timing of CHIKV infection in the bats we studied. Re-capturing bats, particularly during interepidemic periods, would offer valuable insights into infection dynamics and reservoir potential. Finally, experimental infection of the high-seropositive bat species is needed to determine if they develop viremia of adequate magnitude to participate in mosquito transmission. In conclusion, our study strengthens evidence for natural CHIKV exposure in some Old World fruit bat species in West Africa. 

Appendix Additional information for serosurvey of chikungunya virus in Old World fruit bats, Senegal, 2020–2022.

## References

[1] de Souza WM, de Lima STS, Simões Mello LM, Candido DS, Buss L, Whittaker C, et al. Spatiotemporal dynamics and recurrence of chikungunya virus in Brazil: an epidemiological study. Lancet Microbe. 2023;4:e319–29. 10.1016/S2666-5247(23)00033-2 37031687 PMC10281060

[2] Weaver SC, Chen R, Diallo M. Chikungunya virus: role of vectors in emergence from enzootic cycles. Annu Rev Entomol. 2020;65:313–32. 10.1146/annurev-ento-011019-025207 31594410

[3] Althouse BM, Guerbois M, Cummings DAT, Diop OM, Faye O, Faye A, et al. Role of monkeys in the sylvatic cycle of chikungunya virus in Senegal. Nat Commun. 2018;9:1046. 10.1038/s41467-018-03332-7 29535306 PMC5849707

[4] Calisher CH, Childs JE, Field HE, Holmes KV, Schountz T. Bats: important reservoir hosts of emerging viruses. Clin Microbiol Rev. 2006;19:531–45. 10.1128/CMR.00017-06 16847084 PMC1539106

[5] Diallo D, Dia I, Diagne CT, Gaye A, Diallo M. Chapter 4: Emergences of chikungunya and Zika in Africa. In: Higgs S, Vanlandingham DL, Powers AM, editors. Chikungunya and Zika viruses. New York: Academic Press (Elsevier Inc); 2018. p. 87–133 [cited 2024 Jan 8]. https://www.sciencedirect.com/book/9780128118658/chikungunya-and-zika-viruses

[6] Bosco-Lauth AM, Nemeth NM, Kohler DJ, Bowen RA. Viremia in North American mammals and birds after experimental infection with Chikungunya viruses. Am J Trop Med Hyg. 2016;94:504–6. 10.4269/ajtmh.15-0696 26666699 PMC4775881

[7] Stone D, Lyons AC, Huang YS, Vanlandingham DL, Higgs S, Blitvich BJ, et al. Serological evidence of widespread exposure of Grenada fruit bats to chikungunya virus. Zoonoses Public Health. 2018;65:505–11. 10.1111/zph.12460 29575672 PMC7165682

[8] Kading RC, Borland EM, Mossel EC, Nakayiki T, Nalikka B, Ledermann JP, et al. Exposure of Egyptian rousette bats (*Rousettus aegyptiacus*) and a little free-tailed bat (*Chaerephon pumilus*) to alphaviruses in Uganda. Diseases. 2022;10:121. 10.3390/diseases10040121 36547207 PMC9777265

[9] Powers AM, Brault AC, Tesh RB, Weaver SC. Re-emergence of Chikungunya and O’nyong-nyong viruses: evidence for distinct geographical lineages and distant evolutionary relationships. J Gen Virol. 2000;81:471–9. 10644846 10.1099/0022-1317-81-2-471

