# Supplementary material for: Serosurvey of Chikungunya Virus in Old World Fruit Bats, Senegal, 2020–2022
Source: Emerg Infect Dis. 2024 Jul;30(7):1490–2. doi: 10.3201/eid3007.240055 (PMC11210635; doi:10.3201/eid3007.240055)
Supplement: Appendix — Additional information for serosurvey of chikungunya virus in Old World fruit bats, Senegal, 2020–2022. [file 24-0055-Techapp-s1.pdf]

Article DOI: http://doi.org/10.3201/eid3007.240055
*EID cannot ensure accessibility for Supplemental Materials supplied by authors. Readers who have difficulty accessing supplementary content should contact the authors for assistance.*

# Serosurvey of Chikungunya Virus in Old World Fruit Bats, Senegal, 2020–2022

## Appendix

## Materials and Methods

### Bat blood samples collection

Bats were captured during July and December (the rainy season) using mist nets between dusk and dawn in five locations in the Kédougou region, Senegal. After capture, each bat was carefully restrained face-up in a cloth bag to minimize stress. Then, the blood sample was collected by cardiac puncture. Following sample collection, the bats were identified using standard ecologic taxonomic keys and released. Samples were stored in liquid nitrogen during the field to maintain sample integrity. Upon arrival at the laboratory, blood samples were centrifuged at 3,000 rpm for 15 minutes to separate the serum, and then the serum samples were stored at −80°C.

### ELISA to detect antibodies targeting recombinant envelope protein 2 of Chikungunya virus

Our in-house ELISA protocol was based on previously established methods (*1–3*). Briefly, we coated 96-well plates (Cat no. #3690, Corning, USA) with 50 µL/well of a 2 µg/mL solution of chikungunya virus envelope protein 2 (E2) (Cat no. #MBS596329, MyBioSource, USA) in phosphate-buffered saline (PBS, Cat no. #10010–023, GIBCO, USA) overnight at 4°C. We removed the coating solution the next day and blocked the plates with 100 µL/well of 3% non-fat milk in PBS with 0.1% Tween 20 (PBST) for 1 hour at room temperature. To inactivate potential viruses, bat serum samples were heated at 56°C for 1 hour before use. Serial dilutions (1:100) of these serum samples and antibody controls were prepared in 1% non-fat milk PBST. Then, we removed the blocking solution and washed the plates three times with 250 µL/well of 0.1% PBST. Next, we added 100 µL of serial serum dilutions in each well and incubated the

plates for 1 hours at 37°C. Subsequently, the plates were washed three times with 250 µl/well of 0.1% PBST. For antibody detection, we used goat anti-bat IgG-horseradish peroxidase (HRP) conjugate (Cat no. #NB7238, Novus Biologicals, USA) diluted 1:10,000 in 0.1% PBST. We added 100 µL of this secondary antibody solution to each well and incubated for 1 hour. Positive and negative controls were included using a 1:10,000 dilution of goat anti-mouse IgG-HRP conjugate (Cat no. #SA5–10276, Thermo Fisher USA) prepared in the same manner. The plates were then washed three times with 0.1% PBST. After completely drying the plates, we added 100 µL of SIGMAFAST OPD (o-phenylenediamine dihydrochloride; Cat no. #P9187, Sigma-Aldrich, USA) substrate solution to each well. The reaction was allowed to proceed for 10 minutes before being stopped with 100 µl/well of 3M hydrochloric acid. The optical density (OD) was measured at 490 nm (OD490) using a Molecular Devices Versamax Microplate Reader. A sample was considered positive if its OD490 value exceeded the cutoff value, defined as the mean OD of negative controls (uninfected mice) plus three standard deviations. Additionally, we ran the control in the ELISA with only the secondary antibody (goat anti-bat IgG- HRP conjugate), and we did not observe any binding above the cutoff of the assay.

### ELISA cross-reactivity assessment

To evaluate potential cross-reactivity between alphavirus antibodies and other arboviruses with the CHIKV-E2 recombinant protein, we screened polyclonal hyperimmune mouse fluid specific to o'nyong nyong virus, Semliki Forest virus, Venezuelan equine encephalitis virus (VEEV), yellow fever virus, and Uukuniemi virus obtained from the World Reference Center for Emerging Viruses and Arboviruses. Our in-house ELISA using the CHIKV-E2 recombinant protein detected antibodies against o'nyong nyong virus and Semliki Forest virus, suggesting cross-reactivity with these specific alphaviruses. No reactivity was observed with VEEV, yellow fever, or Uukuniemi virus.

### References

1. Amanat F, Stadlbauer D, Strohmeier S, Nguyen THO, Chromikova V, McMahon M, et al. A serological assay to detect SARS-CoV-2 seroconversion in humans. Nat Med. 2020;26:1033–6.

2. Fumagalli MJ, de Souza WM, Romeiro MF, de Souza Costa MC, Slhessarenko RD, Figueiredo LTM. Development of an Enzyme-Linked Immunosorbent Assay To Detect Antibodies Targeting Recombinant Envelope Protein 2 of Mayaro Virus. J Clin Microbiol. 2019;57:e01892–18.

3. Fumagalli MJ, de Souza WM, Espósito DLA, Silva A, Romeiro MF, Martinez EZ, et al. Enzyme-linked immunosorbent assay using recombinant envelope protein 2 antigen for diagnosis of Chikungunya virus. Virol J. 2018;15:112.

**Appendix Table.** Data from serosurvey of Chikungunya virus in Old World fruit bats, Senegal, 2020–2022, showing seropositive bat samples captured in Kédougou region.

| Date | Location | GPS coordinates | ID | Species | Sex | CHIKV OD mean |
|---|---|---|---|---|---|---|
| 26-Oct-2020 | Fadiga | 12°32′59″ N 12°11′55″ W | B13 | *Epomophorus gambianus* | na | 0.09225196 |
| 27-Oct-2020 | Samecouta | 12°36′46″ N 12°8′10″ W | B20 | *E. gambianus* | F | 0.27905196 |
| 27-Oct-2020 | Samecouta | 12°36′46″ N 12°8′10″ W | B21 | *Epomops franqueti* | F | 0.05105196 |
| 28-Oct-2020 | Samecouta | 12°36′46″ N 12°8′10″ W | B25 | *Lissonycteris angolensis* | M | 0.21305196 |
| 28-Oct-2020 | Samecouta | 12°36′46″ N 12°8′10″ W | B29 | *Eidolon helvum* | M | 0.22350196 |
| 30-Oct-2020 | Ngari | 12°38′5″ N 12°15′1″ W | B62 | *E. gambianus* | F | 0.09670196 |
| 24-Nov-2020 | Fadiga | 12°33′00.55″ N 12°11′55.17″ W | B90 | *E. gambianus* | F | 0.19160196 |
| 25-Nov-2020 | Ndebou | 12°30′21.08″ N 12°27′33.48″ W | B99 | *E. gambianus* | M | 0.09285196 |
| 25-Nov-2020 | Ndebou | 12°30′21.08″ N 12°27′33.48″ W | B101 | *E. franqueti* | M | 0.05950196 |
| 25-Nov-2020 | Ndebou | 12°30′21.08″ N 12°27′33.48″ W | B105 | *Micropteropus pusillus* | M | 0.04080196 |
| 26-Nov-2020 | Ndebou | 12°30′21.08″ N 12°27′33.48″ W | B111 | *E. franqueti* | M | 0.14320196 |
| 26-Nov-2020 | Ndebou | 12°30′21.08″ N 12°27′33.48″ W | B112 | *E. gambianus* | M | 0.66280196 |
| -Jun-2021 | Samecouta | 12°36′46″ N 12°8′10″ W | B130 | *E. gambianus* | M | 0.18490196 |
| -Jun-2021 | Samecouta | 12°36′46″ N 12°8′10″ W | B135 | *E. gambianus* | M | 0.39630196 |
| -Jun-2021 | Samecouta | 12°36′46″ N 12°8′10″ W | B144 | *E. gambianus* | M | 0.21855196 |
| -Jun-2021 | Samecouta | 12°36′46″ N 12°8′10″ W | B145 | *E. gambianus* | M | 0.24405196 |
| -Jun-2021 | Samecouta | 12°36′46″ N 12°8′10″ W | B147 | *E. gambianus* | M | 0.14080196 |
| 5-Sep-2021 | Ngari | 12°38′5″ N 12°15′1″ W | B178 | *E. gambianus* | M | 0.11717099 |
| 5-Sep-2021 | Ngari | 12°38′5″ N 12°15′1″ W | B210 | *E. gambianus* | F | 0.55750196 |
| 5-Sep-2021 | Ngari | 12°38′5″ N 12°15′1″ W | B215 | *E. helvum* | M | 0.15015196 |
| 5-Sep-2021 | Ngari | 12°38′5″ N 12°15′1″ W | B216 | *E. gambianus* | M | 0.18795196 |
| 5-Sep-2021 | Ngari | 12°38′5″ N 12°15′1″ W | B217 | *E. gambianus* | M | 0.41480196 |
| 5-Sep-2021 | Ngari | 12°38′5″ N 12°15′1″ W | B219 | *E. gambianus* | F | 0.28045196 |
| 5-Sep-2021 | Ngari | 12°38′5″ N 12°15′1″ W | B221 | *M. pusillus* | M | 0.03655196 |
| 7-Sep-2021 | Ngari | 12°38′5″ N 12°15′1″ W | B227 | *E. helvum* | M | 0.24095196 |
| 7-Sep-2021 | Ngari | 12°38′5″ N 12°15′1″ W | B230 | *E. helvum* | M | 0.12135196 |
| 7-Sep-2021 | Ngari | 12°38′5″ N 12°15′1″ W | B231 | *E. helvum* | M | 0.43235196 |
| 8-Sep-2021 | Samecouta | 12°36′46″ N 12°8′10″ W | B245 | *E. gambianus* | M | 0.47295196 |
| 8-Sep-2021 | Samecouta | 12°36′46″ N 12°8′10″ W | B260 | *E. gambianus* | M | 0.15515196 |
| 8-Sep-2021 | Samecouta | 12°36′46″ N 12°8′10″ W | B262 | *E. gambianus* | M | 0.36100196 |
| 8-Sep-2021 | Samecouta | 12°36′46″ N 12°8′10″ W | B264 | *E. gambianus* | M | 0.11325196 |
| 8-Sep-2021 | Samecouta | 12°36′46″ N 12°8′10″ W | B276 | *E. gambianus* | M | 0.07125196 |
| 8-Sep-2021 | Samecouta | 12°36′46″ N 12°8′10″ W | B289 | *E. gambianus* | M | 0.08770196 |
| 9-Sep-2021 | Samecouta | 12°36′46″ N 12°8′10″ W | B295 | *E. gambianus* | M | 0.12675196 |
| 9-Sep-2021 | Samecouta | 12°36′46″ N 12°8′10″ W | B296 | *E. gambianus* | M | 0.51830196 |
| 9-Sep-2021 | Samecouta | 12°36′46″ N 12°8′10″ W | B304 | *E. gambianus* | M | 0.03450196 |
| 9-Sep-2021 | Samecouta | 12°36′46″ N 12°8′10″ W | B311 | *E. gambianus* | M | 0.08945196 |
| 9-Sep-2021 | Samecouta | 12°36′46″ N 12°8′10″ W | B313 | *E. gambianus* | M | 0.22530196 |
| 9-Sep-2021 | Samecouta | 12°36′46″ N 12°8′10″ W | B314 | *E. gambianus* | M | 0.48400196 |
| 9-Sep-2021 | Samecouta | 12°36′46″ N 12°8′10″ W | B316 | *E. gambianus* | M | 0.05605196 |
| 9-Sep-2021 | Samecouta | 12°36′46″ N 12°8′10″ W | B322 | *E. gambianus* | M | 0.05400196 |
| 9-Sep-2021 | Samecouta | 12°36′46″ N 12°8′10″ W | B327 | *E. gambianus* | M | 0.21035196 |
| 9-Sep-2021 | Samecouta | 12°36′46″ N 12°8′10″ W | B334 | *E. gambianus* | M | 0.10150196 |
| 10-Sep-2021 | Samecouta | 12°36′46″ N 12°8′10″ W | B341 | *E. gambianus* | M | 0.36105196 |
| 10-Sep-2021 | Samecouta | 12°36′46″ N 12°8′10″ W | B342 | *E. gambianus* | M | 0.00780196 |
| 10-Sep-2021 | Samecouta | 12°36′46″ N 12°8′10″ W | B344 | *E. gambianus* | M | 0.26105196 |
| 10-Sep-2021 | Samecouta | 12°36′46″ N 12°8′10″ W | B349 | *E. gambianus* | M | 0.04095196 |
| 11-Sep-2021 | Samecouta | 12°36′46″ N 12°8′10″ W | B353 | *E. gambianus* | M | 0.36125196 |
| 11-Sep-2021 | Samecouta | 12°36′46″ N 12°8′10″ W | B356 | *E. gambianus* | M | 0.96925196 |
| 11-Sep-2021 | Samecouta | 12°36′46″ N 12°8′10″ W | B357 | *E. gambianus* | F | 0.12720196 |
| 11-Sep-2021 | Samecouta | 12°36′46″ N 12°8′10″ W | B361 | *E. gambianus* | M | 0.55580196 |
| 11-Sep-2021 | Samecouta | 12°36′46″ N 12°8′10″ W | B363 | *E. gambianus* | M | 0.38250196 |
| 11-Sep-2021 | Samecouta | 12°36′46″ N 12°8′10″ W | B369 | *E. gambianus* | F | 0.37915196 |

| Date | Location | GPS coordinates | ID | Species | Sex | CHIKV OD mean |
|---|---|---|---|---|---|---|
| 11-Sep-2021 | Samecouta | 12°36′46″ N 12°8′10″ W | B370 | *E. gambianus* | M | 0.31105196 |
| 11-Sep-2021 | Samecouta | 12°36′46″ N 12°8′10″ W | B372 | *E. gambianus* | M | 0.08705196 |
| 11-Sep-2021 | Samecouta | 12°36′46″ N 12°8′10″ W | B375 | *E. gambianus* | M | 0.73960196 |
| 11-Sep-2021 | Samecouta | 12°36′46″ N 12°8′10″ W | B376 | *E. helvum* | M | 0.84650196 |
| 11-Sep-2021 | Samecouta | 12°36′46″ N 12°8′10″ W | B377 | *E. helvum* | M | 0.15215196 |
| 11-Sep-2021 | Samecouta | 12°36′46″ N 12°8′10″ W | B380 | *E. gambianus* | M | 0.29785196 |
| 11-Sep-2021 | Samecouta | 12°36′46″ N 12°8′10″ W | B381 | *E. gambianus* | M | 0.25020196 |
| 11-Sep-2021 | Samecouta | 12°36′46″ N 12°8′10″ W | B387 | *E. helvum* | M | 0.19420196 |
| 11-Sep-2021 | Samecouta | 12°36′46″ N 12°8′10″ W | B388 | *E. helvum* | M | 0.91235196 |
| 11-Sep-2021 | Samecouta | 12°36′46″ N 12°8′10″ W | B389 | *E. helvum* | M | 1.32655196 |
| 11-Sep-2021 | Samecouta | 12°36′46″ N 12°8′10″ W | B394 | *E. gambianus* | F | 0.34235196 |
| 11-Sep-2021 | Samecouta | 12°36′46″ N 12°8′10″ W | B396 | *E. gambianus* | M | 0.09005196 |
| 12-Sep-2021 | Samecouta | 12°36′46″ N 12°8′10″ W | B402 | *E. gambianus* | M | 0.17495196 |
| 12-Sep-2021 | Samecouta | 12°36′46″ N 12°8′10″ W | B403 | *E. gambianus* | M | 0.03795196 |
| 12-Sep-2021 | Samecouta | 12°36′46″ N 12°8′10″ W | B404 | *E. gambianus* | M | 0.09385196 |
| 12-Sep-2021 | Samecouta | 12°36′46″ N 12°8′10″ W | B407 | *E. gambianus* | M | 0.31339727 |
| 12-Sep-2021 | Samecouta | 12°36′46″ N 12°8′10″ W | B409 | *E. gambianus* | M | 0.23734727 |
| 12-Sep-2021 | Samecouta | 12°36′46″ N 12°8′10″ W | B414 | *M. pusillus* | F | 0.26889727 |
| 12-Sep-2021 | Samecouta | 12°36′46″ N 12°8′10″ W | B416 | *E. gambianus* | F | 1.50979727 |
| 12-Sep-2021 | Samecouta | 12°36′46″ N 12°8′10″ W | B418 | *E. gambianus* | M | 0.82364727 |
| 12-Sep-2021 | Samecouta | 12°36′46″ N 12°8′10″ W | B422 | *E. gambianus* | M | 0.18199727 |
| 12-Sep-2021 | Samecouta | 12°36′46″ N 12°8′10″ W | B423 | *E. gambianus* | F | 0.45509727 |
| 12-Sep-2021 | Samecouta | 12°36′46″ N 12°8′10″ W | B424 | *E. gambianus* | M | 0.11659727 |
| 13-Sep-2021 | Ndebou | 12°30′21.08″ N 12°27′33.48″ W | B437 | *E. gambianus* | M | 0.10889727 |
| 13-Sep-2021 | Ndebou | 12°30′21.08″ N 12°27′33.48″ W | B444 | *E. gambianus* | M | 1.22039727 |
| 13-Sep-2021 | Ndebou | 12°30′21.08″ N 12°27′33.48″ W | B445 | *M. pusillus* | M | 0.24119727 |
| 13-Sep-2021 | Ndebou | 12°30′21.08″ N 12°27′33.48″ W | B450 | *M. pusillus* | M | 0.55914727 |
| 13-Sep-2021 | Ndebou | 12°30′21.08″ N 12°27′33.48″ W | B454 | *E. helvum* | F | 0.29589727 |
| 13-Sep-2021 | Ndebou | 12°30′21.08″ N 12°27′33.48″ W | B455 | *M. pusillus* | F | 0.52494727 |
| 13-Sep-2021 | Ndebou | 12°30′21.08″ N 12°27′33.48″ W | B457 | *M. pusillus* | M | 0.14374727 |
| 13-Sep-2021 | Ndebou | 12°30′21.08″ N 12°27′33.48″ W | B458 | *E. gambianus* | M | 0.63804727 |
| 13-Sep-2021 | Ndebou | 12°30′21.08″ N 12°27′33.48″ W | B459 | *E. gambianus* | F | 0.04049727 |
| 13-Sep-2021 | Ndebou | 12°30′21.08″ N 12°27′33.48″ W | B465 | *E. helvum* | F | 0.49589727 |
| 13-Sep-2021 | Ndebou | 12°30′21.08″ N 12°27′33.48″ W | B467 | *E. helvum* | F | 0.22682099 |
| 13-Sep-2021 | Ndebou | 12°30′21.08″ N 12°27′33.48″ W | B469 | *E. helvum* | F | 0.07179727 |
| 13-Sep-2021 | Ndebou | 12°30′21.08″ N 12°27′33.48″ W | B482 | *E. helvum* | F | 0.40562099 |

CHIKV, Chikungunya virus; F, female; GPS, Global Positioning System; ID, identification sample; M, male; na, not available; OD, optical density.

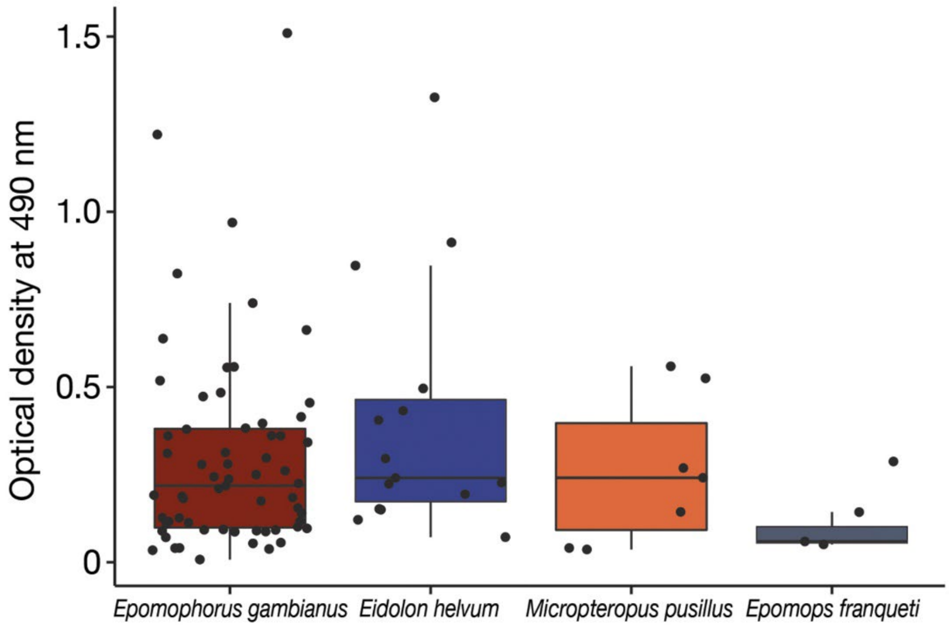


**Appendix Figure.** Measured optical density at 490 nm (OD490) in bat samples positive for antibodies against the envelope 2 recombinant protein of chikungunya virus using in-house ELISA protocol. Four bat species are presented: *Epomophorus gambianus* (n=63), *Eidolon helvum* (n=15), *Micropteropus pusillus* (n=7), *Epodomus franqueti* (n=3). The positive samples from *Lissonycteris angolensis* (n=1, with OD490=0.213) are not presented. The box plot shows the range of data from the median (middle line) to the 25th and 75th percentile (box boundaries), with the whisker lines showing the minimum and maximum values. Individual data points (i.e., OD490 mean) are shown as dots. No statistically significant differences were observed between groups using the unpaired t-test.
